# Parallel Dictionary Reconstruction and Fusion for Spectral Recovery in Computational Imaging Spectrometers

**DOI:** 10.3390/s25154556

**Published:** 2025-07-23

**Authors:** Hongzhen Song, Qifeng Hou, Kaipeng Sun, Guixiang Zhang, Tuoqi Xu, Benjin Sun, Liu Zhang

**Affiliations:** 1 College of Instrumentation & Electrical Engineering, Jilin University, Changchun 130061, China; songhz21@mails.jlu.edu.cn; 2 School of Science, Rensselaer Polytechnic Institute, Troy, NY 12180, USA; hyerichou@163.com; 3 Shanghai Institute of Satellite Engineering, Shanghai 201109, China; kpsun@nuaa.edu.cn; 4 Suzhou Argo Space Technology Co., Ltd., Suzhou 215000, China; zhang_gui_xiang@126.com (G.Z.); xutuoqi@126.com (T.X.); benjin_sun@163.com (B.S.)

**Keywords:** compressed sensing, filter array, spectral recovery, compact spectrometer

## Abstract

Computational imaging spectrometers using broad-bandpass filter arrays with distinct transmission functions are promising implementations of miniaturization. The number of filters is limited by the practical factors. Compressed sensing is used to model the system as linear underdetermined equations for hyperspectral imaging. This paper proposes the following method: parallel dictionary reconstruction and fusion for spectral recovery in computational imaging spectrometers. Orthogonal systems are the dictionary candidates for reconstruction. According to observation of ground objects, the dictionaries are selected from the candidates using the criterion of incoherence. Parallel computations are performed with the selected dictionaries, and spectral recovery is achieved by fusion of the computational results. The method is verified by simulating visible-NIR spectral recovery of typical ground objects. The proposed method has a mean square recovery error of ≤1.73 × 10^−4^ and recovery accuracy of ≥0.98 and is both more universal and more stable than those of traditional sparse representation methods.

## 1. Introduction

In remote sensing, imaging spectrometers are of great interest because of their ability to perform qualitative and quantitative analysis of remote sensing targets. Dispersive devices such as prisms and gratings are used by conventional imaging spectrometers to acquire spectral and spatial information in a push-broom or whisk-broom manner [1,2,3,4]. Owing to practical requirements, researchers have conducted significant research on designing compact imaging spectrometers. The size of the traditional imaging spectrometer is limited by the dispersion method and optical length. In recent years, the planar grating is replaced by a convex/concave grating, which makes the structure compact to meet miniaturization demand [4,5,6].

Research in micro- and nano-optics has enabled effective integration of dispersion systems and facilitated a trend toward lightness and compactness of imaging spectrometers [7]. It is worth noting that the spectral resolution is positively correlated with the optical path length of the system, i.e., applying micro- and nano-optics to dispersive spectrometers is likely to entail sacrificing their spectral resolution. The problem of reduced spectral resolution caused by the reduction in the optical path can be avoided by a push-broom imaging spectrometer with linear variable filters (LVFs). The transmission spectrum of an LVF is a narrow-band Gaussian peak varying in a single direction, and it takes the role of slit and dispersion elements in a conventional spectrometer. Hence, the spectral resolution is significantly limited by the width and number of Gaussian peaks that the LVF can accommodate.

The broad-bandpass film filter array (BFA) is a new spectral information modulation element for hyperspectral imaging that is a substitute for an LVF. The optical-mechanical system of a spectrometer with BFA is similar and allows for miniaturization, but the acquisition mechanism of spectral information of the two are different. The output of a spectrometer using an LVF is the direct-read spectral information, whereas the BFA one output needs to be calculated and unmixed.

Computational spectroscopy was first proposed in 1999 [8] and is fourth-paradigm spectroscopy with the advantages of instrument minimization and higher output SNR [9]. Maximum likelihood analysis has proven that a spectrometer with BFAs can perform unbiased estimation of a target spectrum [10]. An on-chip spectrometer with 195 BFAs has been designed for detecting spectra composed of LEDs. L-curve–Tikhonov regularization, GCV–Tikhonov regularization, and non-negative least-squares methods have been used for effective spectral reconstructions [11,12,13,14]. In recent years, based on emerging materials like quantum dots, various tunable filters have been applied to miniaturize optical spectrometers. A quantum dot colloid with a continuously varying transmission curve was synthesized, enabling least-squares spectral recovery to achieve a resolution of 3 nm in the 390–680 nm range [15]. Thereafter, quantum dots were synthesized and integrated to design a short-wave infrared BFA spectrometer, where a total-variation algorithm was used for spectral reconstruction with an average resolution of 6 nm [16].

After spectral information acquisition, the second most urgent need is spatial information acquisition. Integrating a BFA on a detector entails a lack of spatial imaging capability. The problem of low spatial resolution cannot be solved using a compound optical system to match the BFA. An effective solution is using a broad-bandpass filter line array (BFLA) instead of a dot array and obtaining spatial information in a push-broom manner [17]. The number of integrated filters in the line array is equivalent to the observation frequency. In conventional non-underdetermined solution methods, the number of filters in a BFLA should be greater than or equal to the number of requested spectral bands. However, the number of filters in a BFLA is limited by the processing technology, detector size, and push-broom imaging frame rate, making it impossible to achieve the required observation frequency for sampling information. For hyperspectral imaging, the computational system is linear and underdetermined with a non-unique solution. This issue is alleviated by introducing a sparsity constraint to guarantee a unique solution and implement inversion with insufficient observation [18].

While the introduction of sparsity constraints has enabled the recovery of increased spectral bands, the precision and stability of spectral reconstruction remain constrained by observation frequency and reconstruction algorithms. This limitation underscores the critical need for algorithms to balance high accuracy with universal applicability—especially in error-sensitive domains like medical diagnostics or environmental monitoring, where even minor spectral deviations can lead to erroneous conclusions. For instance, in quantitative analysis of water quality parameters, the sparsity-based reconstruction must mitigate noise amplification from limited sampling, while adapting to diverse water body compositions. Similarly, in agricultural stress detection, the algorithm must reconcile spectral sparsity assumptions with the dynamic reflectance changes of crops under varying environmental conditions, ensuring that reconstruction errors do not obscure subtle physiological signals. Thus, despite the advancements enabled by sparsity constraints, the technology’s utility in real-world applications hinges on its ability to maintain high-fidelity spectral recovery across heterogeneous scenarios.

On the basis of these issues, this paper proposes a parallel dictionary reconstruction and fusion (PDRF) method. To enhance universal applicability across diverse targets, multiple generalized and complete orthogonal systems encompassing a wide range of spectral characteristics are selected as dictionaries for parallel reconstructions. This multi-dictionary design enables the method to adaptively capture intrinsic spectral features of various objects, from typical ground materials to complex mixed scenes. An adaptively weighted fusion strategy is then applied to the individual reconstruction results, dynamically adjusting weights according to the spectral similarity between each dictionary’s output and the target’s intrinsic properties. This adaptive mechanism not only reinforces the stability of composite reconstruction but also elevates precision by leveraging complementary strengths of different dictionaries. The proposed method is validated through simulated spectral reconstruction experiments on typical ground objects and extended to heterogeneous scenarios involving mixed materials, demonstrating its versatility. Comparative analyses against typical methods utilizing single complete orthogonal systems further verify that PDRF exhibits superior accuracy and stability, particularly in handling targets with complex spectral signatures, thereby embodying both broad adaptability and fine-grained precision in spectral reconstruction.

The rest of this paper is structured as follows. Section 2 analyzes the principle and advantages of the push-broom imaging spectrometer with a broad-bandpass filter line array (IS-BFLA) and establishes the corresponding discretized mathematical model for hyperspectral imaging. Section 3 proposes the parallel dictionary reconstruction and fusion (PDRF) method, elaborating on its implementation process in detail, including dictionary selection, parallel reconstruction, result screening, and adaptive fusion. Section 4 introduces the testing environment and evaluation methods, covering the dataset, dictionary types selected, and metrics for assessing reconstruction performance. Section 5 presents the testing and analysis of PDRF, including simulated spectral reconstruction experiments on typical ground objects, and compares the results with those of other methods to verify the superiority of the proposed method. Finally, Section 6 summarizes the conclusions of this study.

## 2. Principle and Modeling of IS-BFLA Hyperspectral Imaging

### 2.1. Modeling of IS-BFLA

**Figure 1:**
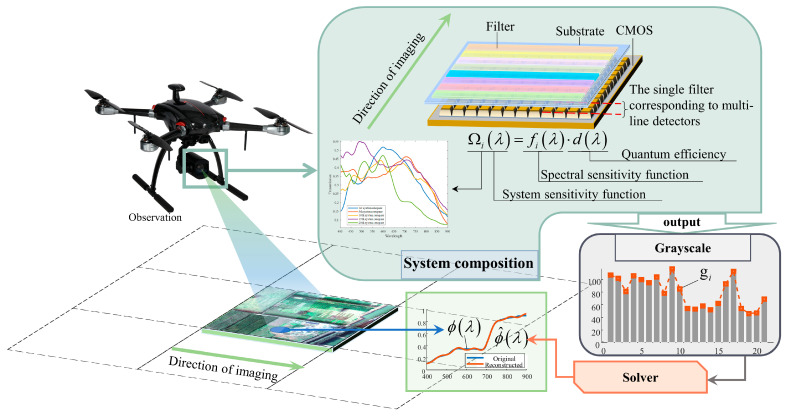
Principle of the IS-BFLA.

The principle of the IS-BFLA is shown in Figure 1. Its main components include the optical system, the BFLA, and CMOS detector with the output circuit. The IS-BFLA is more suitable for remote sensing on motion platforms such as in aviation or spaceflight. First, during push-broom imaging, the object spectrum passes sequentially through all the broad-bandpass film filters in the BFLA. The filters in the BFLA have broad-bandpass transmittance, which significantly improves the optical energy for imaging. Second, the single filter corresponds to the multi-line pixels in the detectors. Time delay integration (TDI) technology can be used to improve the imaging quality further. Therefore, the transmitted intensity in the IS-BFLA is upgraded in the same exposure time as in the conventional spectrometer. Theoretically, the IS-BFLA more easily achieves compatibility between high spatial and high spectral resolution.

The filters in a BFLA have unique spectral transmission functions. The object spectra are weighted by the spectral transmission functions of the filters, and the light energy is recorded by the corresponding detector pixels. The output gray value *g_i_* can be treated as a dual integration of wavelength and exposure time and can be expressed as

(1) $$g_{i}=\int_{t}\int_{\lambda }f_{i}\left(\lambda \right)\cdot d\left(\lambda \right)\cdot \phi \left(\lambda ,t\right)\cdot d\lambda \cdot dt\;,$$

where $f_{i}\left(\lambda \right)$ is the joint spectral sensitivity function of the *i*th filter and the optical system, $d\left(\lambda \right)$ is the detector quantum efficiency function, and $\phi \left(\lambda \right)$ is the target spectral function. Equation (1) can be simplified by letting

(2) $$\Omega_{i}\left(\lambda \right)=f_{i}\left(\lambda \right)\cdot d\left(\lambda \right)\;,$$

where Ω*_i_* (*λ*, *t*) is the spectral sensitivity function of the system obtained by modulating the *i*th filter. Ideally, the push-broom motion is fast and stable, and the incident energy of the target spectrum into different filters can be considered constant during the imaging time. Therefore, *ϕ*(*λ*, *t*) in Equation (1) is a non-time-varying signal. For the *λ* integral term, the rectangular integral approximation is adopted to discretize the linear system. Equation (1) can be expressed in matrix form as

(3) $$\Delta \lambda \cdot \Delta t\left[\begin{array}{ccccc} \Omega_{1,1} & \ldots & \Omega_{1,n} & \ldots & \Omega_{1,N}\\ \vdots & \ddots & \vdots & \ddots & \vdots \\ \Omega_{m,1} & \ldots & \Omega_{m,n} & \ldots & \Omega_{m,N}\\ \vdots & \ddots & \vdots & \ddots & \vdots \\ \Omega_{M,1} & \ldots & \Omega_{M,1} & \ldots & \Omega_{M,1} \end{array}\right]\left[\begin{array}{c} \phi_{1}\\ \vdots \\ \phi_{n}\\ \vdots \\ \phi_{N} \end{array}\right]=\left[\begin{array}{c} g_{1}\\ \vdots \\ g_{m}\\ \vdots \\ g_{M} \end{array}\right]\;,$$

where the time integration term ∆*t* and spectral integration term ∆λ can be directly introduced into the spectral sensitivity functions. The IS-BFLA model can be further simplified to obtain

(4) Ωϕ=g ,

where **Ω** $\in R^{M\times N}$ is the system spectral sensitivity matrix, $\phi \in R^{N\times 1}$ is the object spectrum vector, and ***g*** $\in R^{M\times 1}$ is the vector with the imaging grayscale value. The spectral reconstruction corresponds to solving ϕ from Equation (4) with known ***g*** and **Ω**.

If the frequency of observations, namely, the number of filters in a BFLA, is greater than or equal to the number of required spectral bands, the system of linear equations is non-underdetermined. After the condition number of the system is calculated and the anti-interference ability is optimized, high-precision spectral reconstruction can be directly performed using convex solving methods with the *l*_2_-norm as the objective function, such as with least squares or regularization, and the hyperspectral remote sensing data cube with a 2D spatial image and 1D spectrum can be obtained.

### 2.2. Compressed-Sensing Spectral Reconstruction Method for IS-BFLA Hyperspectral Imaging

When the observations of the IS-BFLA are insufficient, the reconstruction system is underdetermined, resulting in the problem of non-unique solutions. Therefore, the sparsity constraint is introduced to ensure unique solutions and conduct reliable hyperspectral recovery. The target spectrum can be directly reconstructed using the sparse representation if the spectrum is inherently sparse, e.g., the target signal is a Gaussian peak in a specific range. Unfortunately, the reflection or emission spectra of objects are generally in a continuous non-zero form. Hence, the sparse transformation needs to be introduced into the current system. After calculating the sparse solution, the target spectrum is indirectly calculated by combining the sparse solution with the sparse transformation.

The dictionary $A\in R^{N\times L}$ is designed for implementing the sparse transformation and serves as the sparse constraint. We let

(5) Ax=ϕ,

where $x\in R^{L\times 1}$ is a k-sparse vector (k << L). Substituting Equation (5) into Equation (4) gives

(6) $$\begin{array}{c} \Theta x=g,\\ s.t.\;\Theta =\Omega A. \end{array}$$

In compressive sensing theory, the system sensitivity matrix Ω is also called the observation matrix, and the matrix $\Theta \in R^{M\times L}$ is called the compressive sensing matrix. The compressed sensing matrix obtained by the sparse transformation is still underdetermined, but the amount of solved data is reduced by constraining the selection of sparse variables. Therefore, selecting the correct column vectors in the compressed sensing matrix and the accuracy of the corresponding coefficients become the key factors for high-precision spectral reconstruction.

The sparse representation constrained by the l_0_-norm is a nonconvex problem, i.e., a non-deterministic complete polynomial. Therefore, the l1-norm is applied as an alternative to the l0-norm for the relaxation optimization solution to ensure that the objective function is convex. Specific solution methods include convex optimization and greedy algorithms.

There are three common methods to construct dictionaries. The first method is to select the general transform system, such as a discrete Fourier transform (DFT), discrete cosine transform (DCT), discrete wavelet transform (DWT), or other complete orthogonal system. Most spectral signals are sparse after the transformation. The second method is transform domain training based on the spectral database, i.e., dictionary learning, which can guarantee the sparse transform of any ground objects in the spectral database. The third method is the augmented dictionary, which is based on the former two methods and involves concatenating a variety of dictionaries. The aim of concatenation is to construct a richer transform set for selecting dictionary column vectors and perform the recovery from the local to the global perspective.

To ensure that the solution is non-singular when performing sparse solutions, the observation frequency is usually required to be two times the sparsity k [19]. Therefore, the number of dictionary column vectors chosen is limited by the frequency of observations.

## 3. Parallel Dictionary Reconstruction and Fusion Method

**Figure 2:**
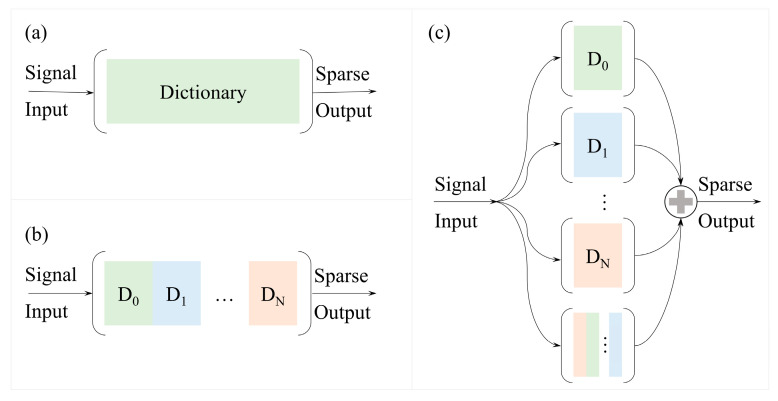
Sparse representation using multiple dictionary configurations. (**a**) Sparse representation based on a single dictionary type, including orthogonal basis and dictionary learning. (**b**) Augmented dictionary (A-dict) formed by concatenating multiple dictionary types. (**c**) The PDRF method.

**Figure 3:**
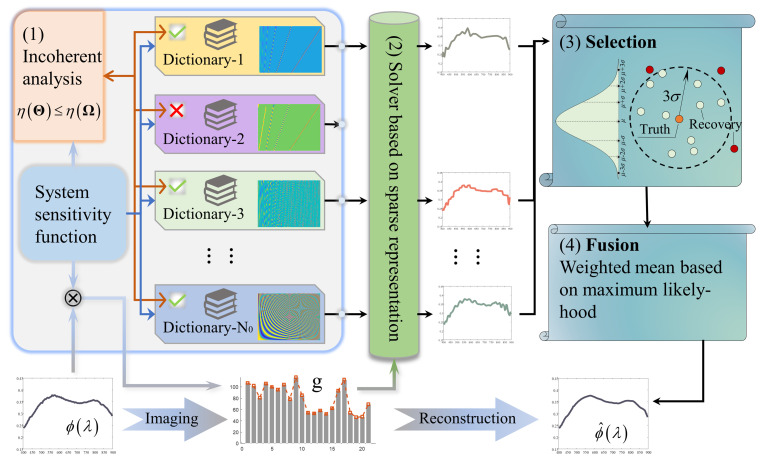
Spectral reconstruction based on the PDRF method.

The PDRF method is proposed by drawing on the idea of series and parallel architecture in circuits, and the difference between this method and traditional sparse representation is illustrated in the Figure 2. This method includes (1) selection of dictionaries, (2) parallel reconstruction of multiple dictionaries, (3) screening of reconstructions, and (4) adaptive fusion of the results, with all these components and the specific methodological elements illustrated in Figure 3.

First, the dictionary set constructed for the current spectral reconstruction contains a variety of complete orthogonal systems, and the augmented dictionary is formed by combining various orthogonal systems. To determine whether the current dictionary can perform effective recovery, the relevant criteria in compressed sensing are introduced to analyze and filter each dictionary in the dictionary set. References [20,21,22] indicate that the restricted isometry property (RIP) and incoherence principle are equivalent and powerful criteria for the feasibility of sparse reconstruction, and low coherence ensures that measurements are “spread out” in the sparsity domain, improving reconstruction. That could serve as a priori judgments for determining whether effective compressive sensing reconstruction can be achieved. The calculation of the coherence is aimed at the compressive sensing matrix and is specifically expressed as

(7) $$\eta \left(\Theta \right)=\underset{1\leq n1<n2\leq N}{\max}\frac{\left|\langle \Theta_{n1},\Theta_{n2}\rangle \right|}{{\left\|\Theta_{n1}\right\|}_{2}{\left\|\Theta_{n2}\right\|}_{2}}\;,$$

where $\Theta_{n}$ is the column vector of the compressive sensing matrix. The judgment criterion is to calculate the coherence of the observation matrix and compare it with that of the compressive sensing matrix. If the coherence increases, the observation matrix is eliminated, so as to guarantee the sparse reconstruction of unknown spectral curves.

Second, the compressed sensing matrix is constructed for the filtered dictionary, and the sparse vector corresponding to each dictionary is calculated using the l1 norm. Then, the reconstructed spectrum is calculated to form the reconstructed spectrum set.

Third, secondary screening is carried out for the reconstructed spectrum corresponding to each selected dictionary. On the condition that the spectral recovery is accurate, the reconstructed spectra corresponding to multiple dictionaries will be identical. Thus, the mean and variance of all reconstructed spectra are calculated in the spectral bands, and the gross errors are judged according to the 3σ criterion. The reconstructed spectra containing gross errors are eliminated, and the secondary screening process is repeated until all reconstructed spectra meet the 3σ criterion. The filtered set of reconstructed spectra can be written in matrix form:

(8) $$\hat{\phi }=\left[\begin{array}{ccccc} {\hat{\phi }}_{1,sel-1} & \ldots & {\hat{\phi }}_{1,sel-n_{1}} & \ldots & {\hat{\phi }}_{1,sel-N_{1}}\\ \vdots & \ddots & \vdots & \ddots & \vdots \\ {\hat{\phi }}_{n,sel-1} & \ldots & {\hat{\phi }}_{n,sel-n_{1}} & \ldots & {\hat{\phi }}_{n,sel-N_{1}}\\ \vdots & \ddots & \vdots & \ddots & \vdots \\ {\hat{\phi }}_{N,sel-1} & \ldots & {\hat{\phi }}_{N,sel-n_{1}} & \ldots & {\hat{\phi }}_{N,sel-N_{1}} \end{array}\right]={\left[\begin{array}{c} {\hat{\phi }}_{sel-1}^{T}\\ \vdots \\ {\hat{\phi }}_{sel-n1}^{T}\\ \vdots \\ {\hat{\phi }}_{sel-N_{1}}^{T} \end{array}\right]}^{T}\;.$$

The element ${\hat{\phi }}_{n,sel\_n1}$ in the matrix is the estimated reconstruction of the *n*th spectral band of the sel_n1 dictionary obtained after screening, and $N_{1}$ reconstructed spectra are selected in the formula. Gross errors are eliminated by secondary screening processes to ensure the global accuracy of the reconstruction.

Finally, the reconstructed spectra after secondary screening are fused adaptively in correspondence with the spectral band. Under-sampling observations still results in loss of detail for the signal, and the degree of global and detail loss is different when using each type of dictionary. Thus, weighted fusion of all the reconstructions is carried out. The reconstructed spectrum is estimated without bias on the basis of maximum likelihood, and the weighted average is calculated from the spectral bands; that is, the weighted values in different spectral bands of the same reconstructed spectrum are individual. The weighting formula is

(9) $$w_{n,sel-n_{1}}=\exp\left(\frac{{\left({\hat{\phi }}_{n,sel-n_{1}}-\mu ({\hat{\phi }}_{n})\right)}^{2}}{\sigma^{2}({\hat{\phi }}_{n})}\right)\;,$$

where $\mu ({\hat{\phi }}_{n})$ and $\sigma^{2}\left({\hat{\phi }}_{n}\right)$ are unbiased mean and variance estimates, respectively. Thus, the weighted matrix w can be obtained. Finally, the reconstructed spectrum with weighted mean fusion is

(10) ϕ^end=wTϕ^wΣ,

where Σ is the row vector summation matrix of the weighted matrix. Through adaptive parameter weighting, multiple reconstructed spectra are adaptively fused in correspondence with the bands.

Selecting the column vectors of multiple dictionaries for the same output signal increases the number of selected column vectors, which weakens the limitation placed by the observation frequency on the column vector selections of the augmented dictionary. On the basis that the target spectrum is the only accurate solution, similar or consistent solutions should exist among the solutions of multiple dictionaries selected for reconstruction. Through error determination, interference is removed, and adaptive fusion is performed, thus ensuring the stability and accuracy of the reconstruction results.

## 4. Testing Environment and Evaluation Method

### 4.1. Dataset Introduction

**Figure 4:**
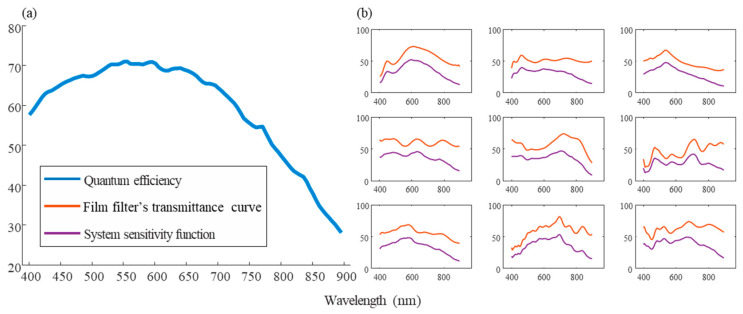
System sensitivity function. (**a**) Quantum efficiency of CMOS. (**b**) Partial spectral response curve in the system.

In a previous study, 20 film filters and a large-area CMOS detector were selected and integrated into a BFLA [23]. The transmittances of BFLAs were measured and combined with the quantum efficiency curve to obtain spectral sensitivity functions of the system as shown in Figure 4. These BFLAs are all transmissive within the 400–900 nm spectral range. However, their transmission efficiencies vary at different wavelengths, which enables the encoding of spectral information at different wavelengths.

To verify the research method proposed in this paper, the terrestrial object spectral dataset from the USGS Digital Spectral Library Version 7 was selected [24]. It included rocks, soils, natural mineral mixtures, various plant types with different backgrounds, and so on. Since this study corresponded to the spectral image reconstruction method within the 400–900 nm imaging spectral range, the dataset was filtered to ensure that it contained valid data within the research spectral range for support. Finally, 991 types of terrestrial object spectral data were obtained. Meanwhile, the data were preprocessed so that the spectral resolution of the spectral data was 5 nm and the value range was [0, 1].

### 4.2. Dictionary Selection

The PDRF framework studied in this paper adopted traditional orthogonal systems without prior learning as the main approach for spectral recovery. The dictionary types included Discrete Cosine Transform (DCT), Coiflet wavelets (coif *N*), Daubechies wavelets (db *N*), Fejér–Korovkin wavelets (fk *N*), Symlet wavelets (sym *N*), etc. [25,26,27]. In view of the characteristics of wavelet bases, the maximum order N of the wavelets was determined based on the existing 100 spectral bands, and the wavelet orthogonal basis was constructed using this maximum order [25,26].

It is worth noting that the Orthogonal Matching Pursuit (OMP) algorithm was still implemented based on least squares internally. If the sparsity was greater than the number of observations, the solution method needed to be switched from least squares to Singular Value Decomposition (SVD), and the uncertainty in its solution accuracy would increase. Therefore, it was required that in any wavelet dictionary, there existed 20 vectors (equal to the number of observations) such that the sparsity of the signal after their summation was 0, thereby achieving a support effect consistent with the length of the signal vector.

**Figure 5:**
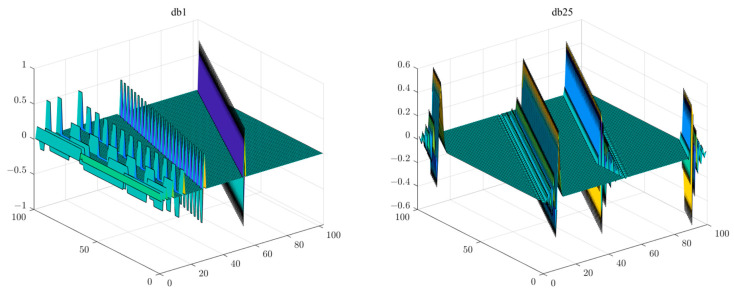
The support vectors of db1 wavelet and db25 wavelet: As the order N increases, the scale supported by a single vector in the wavelet orthonormal system becomes shorter. Under the number of observations in an effective compressive sensing system, if the wavelet signal cannot cover the entire signal range with a limited number of orthogonal vectors (no more than the number of observations), there will be spectral signals that cannot be recovered.

Taking the db1 wavelet and db25 wavelet in Figure 5 as examples, it can be clearly seen that the length of the signal supported by db1 wavelet vectors of the same order was superior to that of db25. Moreover, since db25 could not have 20 vectors whose sum was equivalent to the length of the signal, it could only achieve this through higher sparsity, which was not feasible in the current system. Thus, the db1 wavelet could be selected as a dictionary, while db25 could not.

Therefore, the finally selected dictionaries included coif1~coif4, db1~db13, fk4, fk6, fk8, fk18, fk22, sym4~sym8, and sym10~sym13 (Symlet wavelets were derived from Daubechies (db) wavelets; among them, db1~db3 were consistent with sym1~sym3 and thus were not reused), totaling 31 types of wavelet dictionaries.

There were a total of 32 types of individual orthonormal system dictionaries. Through the concatenation of the aforementioned dictionaries, the 33th dictionary—the augmented dictionary (A_dict)—was constructed. To verify its performance in comparison with dictionary learning types, the KSVD algorithm was introduced for dictionary learning, forming the 34th dictionary [28]. To control algorithm-related factors, a general reconstruction algorithm based on the rapid estimation of orthogonal matching pursuit representation algorithm was selected [28]. Meanwhile, to verify the effects of 3σ filtering and Gaussian weighting, an additional comparison term was added to directly output the mean values of each orthonormal system dictionary (PDRF with mean).

### 4.3. Evaluation Methods for Reconstruction Results

The core goal of spectral reconstruction is to make the reconstructed spectrum as close as possible to the true spectrum, and its essence lies in minimizing the error between the two. The coefficient of determination $R^{2}$ peak signal-to-noise ratio (PSNR) is used to evaluate the accuracy of spectral recovery. In statistics, $R^{2}$ is adopted to detect the variance between the reconstructed data and the original data from a global perspective and evaluate the strength of the linear relationship. The formula for $R^{2}$ is

(11) $$R^{2}=1-\frac{\sum_{i=1}^{n}{\left(\phi \left(\lambda_{i}\right)-\hat{\phi }\left(\lambda_{i}\right)\right)}^{2}}{\sum_{i=1}^{n}{\left(\phi \left(\lambda_{i}\right)-\frac{1}{n}\sum_{j=1}^{n}\phi \left(\lambda_{j}\right)\right)}^{2}}\;,$$

where $\phi \left(\lambda \right)$ is the true spectral value, while $\hat{\phi }\left(\lambda \right)$ is the spectral value reconstructed after observation. Spectral recovery is better verified as R^2^ approaches 1. The calculation formula of PSNR is expressed as

(12) $$PSNR=10\log_{10}\left(\frac{{\left(MAX\left(\phi \left(\lambda \right)\right)\right)}^{2}}{MSE}\right),\;s.t.\;\mathrm{MSE}=\frac{1}{n}\sum_{i=1}^{n}{\left(\phi \left(\lambda_{i}\right)-\hat{\phi }\left(\lambda_{i}\right)\right)}^{2},$$

where $MAX\left(\phi \left(\lambda \right)\right)$ is the maximum value of the spectral data, and MSE measures the overall deviation between the reconstructed spectrum and the true spectrum. The higher the PSNR value, the smaller the error between the reconstruction results and the true values, which directly reflects the fidelity of the reconstruction.

## 5. Testing and Analysis of PDRF

**Figure 6:**
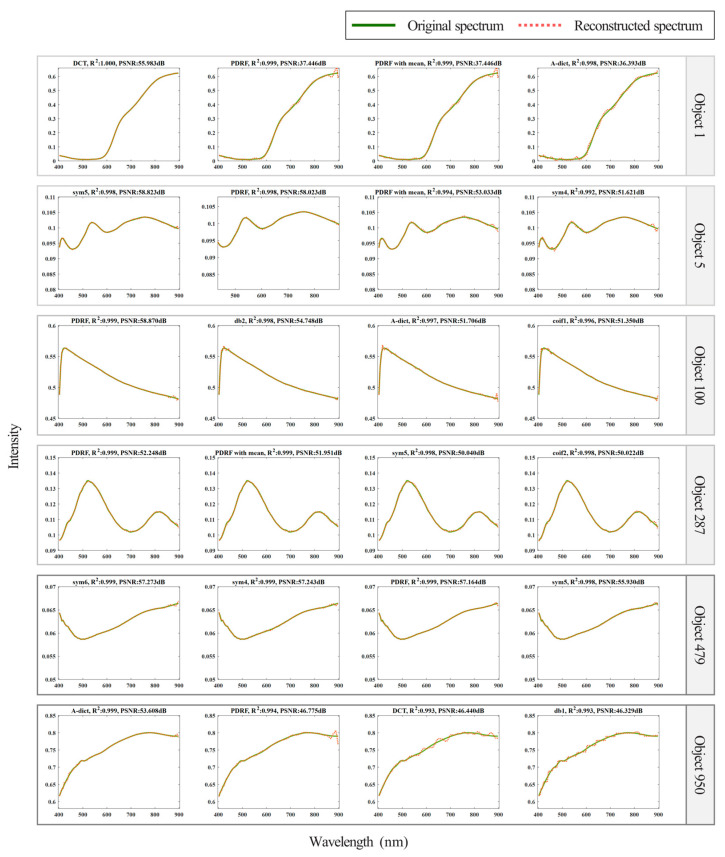
Partial results of spectral reconstruction. The reconstructed objects in the figure are two of 21 objects. (1) to (10) and (11) to (20) are reconstructions of ground objects 4 and 7, respectively, based on 10 dictionaries.

For the analysis of reconstruction performance, six objects were selected for analysis. Based on their recovery accuracy, the top four with the highest accuracy were chosen and plotted in Figure 6. It is easy to observe from the figure that under the same signal length, Object 1 and Object 950 had a wider response range compared with the other four ground objects. Their rising intervals were gentle, with no obvious spectral bands; thus, within a single dictionary, DCT achieved higher recovery accuracy. Object 5, Object 100, Object 287, and Object 479 all exhibited high gradient changes within a shorter signal length. Therefore, wavelets with short support structures achieved better spectral recovery performance for these ground objects.

A-Dict has both global variables provided by the DCT and local variables provided by the wavelet. The variable type contains low-frequency and high-frequency information, so A-Dict has richer sparse vectors for selection. With an effective combination of global and local variables and low- and high-frequency information, A-Dict has more advantages in the global and local aspects of spectral reconstruction, as reflected in the recoveries of object 950. A-Dict also has a great variety of vectors and contains strongly correlated column vectors owing to the concatenation of dictionaries, resulting in increased error in solving sparse vectors. It is worth noting that due to containing a great variety of vectors, A-Dict may contain strongly correlated column vectors, which will lead to increased errors when solving for sparse vectors in Object 858.

**Figure 7:**
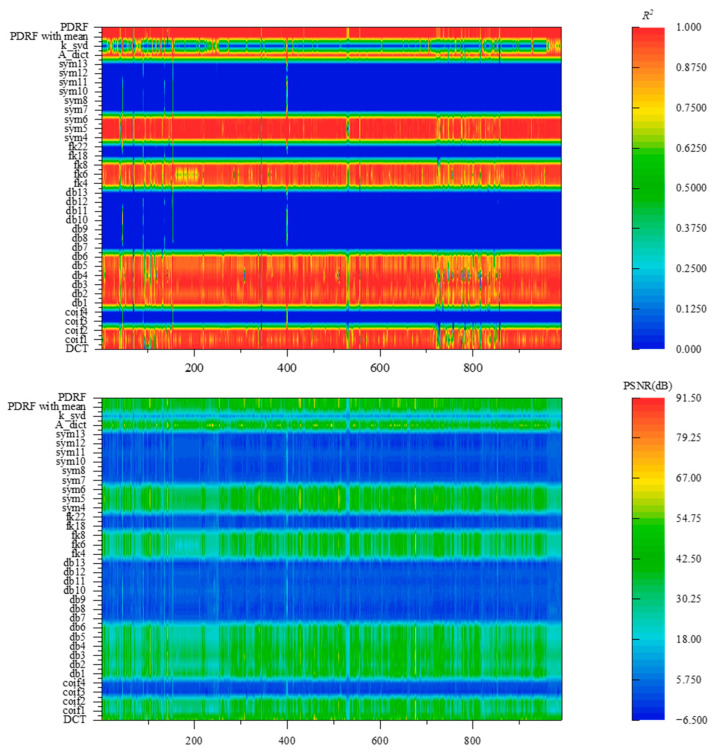
Evaluation of spectral recovery accuracy for multiple types of ground objects. PDRF exhibits high spectral recovery accuracy and has good generalizability for spectral recovery across different targets.

Figure 7 uses R^2^ and PSNR as evaluation metrics to show the spectral recovery performance of different targets under different dictionaries and PDRF. First, it can be observed that applying a single complete orthogonal system as a dictionary suffers from the problem of non-universal applicability, i.e., the target spectrum may be non-sparse under one dictionary transformation but sparse under another. Second, dictionary learning can ensure that all target spectra in the database are sufficiently sparse under this transformation. However, considering the incompleteness of the database and the complexity of actual spectra, issues such as incompleteness and the coherence of the observation system’s response function are unavoidable, which leads to a decrease in the accuracy of target spectral reconstruction.

Because multi-dictionary parallel reconstructions are adopted, the PDRF method is inevitably affected by the recovery accuracy of each dictionary. The global accuracy of the reconstructed spectrum can be estimated with the 3σ criterion, and the adaptive spectral segment fusion provides a second correction of the reconstruction using various dictionaries for the details. In stark contrast to the method, in addition to what has been analyzed above, there is also the result obtained without screening and by directly calculating the mean—PDRF with mean. It obviously exhibits non-universality for some targets, as well as an increase in spectral recovery errors.

The major advantage of PDRF is its ability to reconstruct stably and accurately and its suitability for various ground objects. It is worth noting that the variance of the recovery MSE of the proposed method is one order of magnitude lower than that of other methods for various ground objects. The fusion of multiple dictionary reconstructions enables PDRF to reconstruct from the local to the global scale compared with using a single dictionary. The recovery accuracy of PDRF is stable with a coefficient of determination $R^{2}$ above 0.98, while that of A-Dict is above 0.86. Therefore, the proposed method, PDRF, achieves more stable and precise reconstruction for the compressed sensing system consisting of the IS-BFLA.

## 6. Conclusions

This paper addressed the problem of acquiring hyperspectral information with under-sampling using a spectrometer with a few BFLAs. A compressed sensing system was constructed to solve the problem of non-unique solutions of the underdetermined system using sparsity constraints. A method based on parallel dictionary reconstruction and fusion was proposed. The proposed method focuses on solving two problems: one is that selecting a single orthogonal system as a dictionary has insufficient sparsity, and the other is that the insufficient observation frequency of the system limits the reconstruction accuracy. By constructing the independent dictionaries, A-Dict, dictionary learning with k-svd and PDRF, enables 991 kinds of ground object spectra to be reconstructed respectively and successfully. This verifies the stability and high precision of reconstructions using PDRF, which lays a foundation for subsequent actual reconstruction experiments and provides a new idea for compressed-sensing reconstruction. In future research, more stringent RIP conditions will be introduced into the research, and a hyperspectral imaging experiment will be carried out with the computational imaging spectrometer employing 20 BFLAs.

## Data Availability

Dataset available on request from the authors.
